# A Simple Difficulty

**Published:** 1889-06-01

**Authors:** 


### Within the Wards.

### A SIMPLE DIFFICULTY.

The word "tumour" has an alarming sound to many uninstructed persons. It is a very innocent word, and is often used in quite an innocent sense by medical men. It means no more than a "swelling," although, of course, a "swelling" may be as harmless as a wen or as dangerous as a cancer. The story now to be related will show how easily and harmlessly a tumour may sometimes be produced, and what simple means may be sufficient for its cure. J. B., a young man of twenty-nine, applied to a country surgeon about the middle of last February, complaining of a swelling underneath the left jaw. The surgeon examined, and found the swelling, which was then no larger than a horse-bean. But J. B. said that its present size was nothing; for every time he began to eat, or even smelt a dish which was more than usually appetising, up rose the tumour, as if it were a living thing, and became in a few minutes almost as large as his fist. J. B. was no physiologist, but neither does he seem to have been a fool. No doubt he often wondered what was the matter with his erratic gland, but he was a long time in growing sufficiently alarmed to fly to a doctor. He endured the swelling and its subsidence more or less patiently for four years. Then he was persuaded to consult a medical man. The surgeon happened to be one who had not forgotten either his anatomy or his physiology, and he at once decided what must be the matter. Certain glands beneath the jaw, he knew, secrete saliva, and they secrete it most when it is most needed, that is, when their owner is eating, and needs saliva to moisten his food and to begin digestion. And one of these glands has many ducts, which unite in forming one canal of very considerable size, called Wharton's duct, and this duct is the large main conduit by which the saliva is poured into the mouth. "There must be a 'block' in Wharton's duct," argued the country surgeon. "Every time the patient eats, or would like to eat, the gland begins to do its duty, and to secrete saliva, but no matter how much saliva is secreted, it cannot flow into the mouth because of the tight 'block' in Wharton's duct. What, then, is to be done? Find out what the nature of the block is, and whether it can be removed." Getting the help of a professional brother, the doctor soon discovered that the "horse-bean" was a calculus, or stone, which had formed in Wharton's duct, exactly as it might have formed in the bile duct, in the kidney, or in the bladder. With a little trouble the stone was removed, and a complete cure effected. The gland, though accustomed to swell for four years, no sooner got a free outflow for its secretion than it ceased to enlarge, and became exactly like its fellow of the opposite side. The case is very simple, but affords an instructive lesson on the value of anatomical and physiological knowledge in medical practice.

